# Systematic and mechanistic analysis of AuNP-induced nanotoxicity for risk assessment of nanomedicine

**DOI:** 10.1186/s40580-022-00320-y

**Published:** 2022-06-09

**Authors:** Euiyeon Lee, Minhyeong Lee, San Kwon, Jongpil Kim, Youngeun Kwon

**Affiliations:** 1 Department of Biomedical Engineering, Dongguk University, Seoul, 04620 Korea; 2 Department of Chemistry and Chemical Biology, Rutgers University, Piscataway, NJ 08854 USA; 3 Department of Chemistry, Dongguk University, Seoul, 04620 Korea

**Keywords:** Gold nanoparticles, Mechanism of nanotoxicity, Physicochemical parameters, Nano-bio interaction, Nanomedicine, Risk assessment

## Abstract

For decades, nanoparticles (NPs) have been widely implemented in various biomedical fields due to their unique optical, thermal, and tunable properties. Particularly, gold nanoparticles (AuNPs) have opened new frontiers in sensing, targeted drug delivery, imaging, and photodynamic therapy, showing promising results for the treatment of various intractable diseases that affect quality of life and longevity. Despite the tremendous achievements of AuNPs-based approaches in biomedical applications, few AuNP-based nanomedicines have been evaluated in clinical trials, which is likely due to a shortage of understanding of the biological and pathological effects of AuNPs. The biological fate of AuNPs is tightly related to a variety of physicochemical parameters including size, shape, chemical structure of ligands, charge, and protein corona, and therefore evaluating the effects of these parameters on specific biological interactions is a major ongoing challenge. Therefore, this review focuses on ongoing nanotoxicology studies that aim to characterize the effect of various AuNP characteristics on AuNP-induced toxicity. Specifically, we focus on understanding how each parameter alters the specific biological interactions of AuNPs via mechanistic analysis of nano-bio interactions. We also discuss different cellular functions affected by AuNP treatment (e.g., cell motility, ROS generation, interaction with DNA, and immune response) to understand their potential human health risks. The information discussed herein could contribute to the safe usage of nanomedicine by providing a basis for appropriate risk assessment and for the development of nano-QSAR models.

## Introduction

A wide variety of nanotechnology-based solutions for various applications have been developed over the past decades by exploiting the unique optical, electronic, and thermal properties of nanomaterials [1–5]. Particularly, nanotechnology has greatly contributed to the development of biomedical applications including drug carriers, contrast agents for bioimaging, and therapeutic agents for the treatment of diseases [6, 7]. Nanomedicine recently became an integral part of the healthcare industry and gold nanoparticles (AuNPs) have attracted particular interest among various nanomaterials, as they offer distinct advantages including their relatively low cytotoxicity, high colloidal stability, and tunability [8, 9]. AuNPs can be easily fabricated in different sizes ranging from 1 to more than 100 nm, as well as in various shapes such as spheres, rods, stars, cubes, etc. Furthermore, AuNPs can be easily modified with various molecules to introduce ligands or functional groups of interest, thereby endowing AuNPs with excellent biocompatibility, targeting, and drug delivery capabilities.

There have been quite a few recent breakthroughs in nanomedicine that offer innovative solutions to overcome the limitations of current medical approaches. Particularly, nanotechnology has been instrumental for the advancement of cancer research. For example, AuNPs modified with cancer cell-specific ligands or antibodies have been used to target specific malignant cells to deliver anti-cancer drugs [10]. Additionally, plasmonic AuNPs are often used for the imaging and thermal killing of cancerous cells, as they efficiently absorb photons and convert photonic energy into thermal energy. Moreover, cationic AuNPs are used as a gene carrier for the treatment of various disorders. For example, AuNPs have been used to deliver cardiac reprogramming factors and promote the recovery of cardiac function in patients with cardiac disease [11]. Furthermore, recent developments in AuNP-mediated nanoelectronics have opened new opportunities in the treatment of aging-associated diseases by enhancing in vivo cellular reprogramming efficiency, in which AuNP served as gene delivery vehicle as well as nanoelectrode to promote the activation of neuron-specific gene transcription.

Basic research on the implementation of nanotechnology is currently starting transition toward clinical trials [12, 13]. Nanomedicine research has demonstrated the enormous therapeutic potential of nanotechnology. However, there are few examples of AuNPs being actively investigated in clinical trials and no AuNP-based nanomedicines have thus far been approved by the FDA [14]. This translational pathway has been partly halted by the unpredictability of the hazards and true advantages of nanomedicines [15]. As such, understanding the biological effects of various nanoparticles (NPs) and their consequences has become an important research topic in the context of proper risk assessment and risk minimization [16]. Current research has largely focused on how various physicochemical parameters such as size, shape, chemical structure of ligand, charge, and protein corona alter the biological interactions of NPs both in vivo and in vitro. While in vivo model systems provide insights into the potential effects of NPs on the human body, individual effects on various biological functions cannot be easily evaluated as biological systems are tightly interrelated. Additionally, estimating the long-term or cumulative effects of these materials is critical to assess/manage their potential risks; however, such estimations cannot be easily made based on our current understanding of NP interactions. More importantly, there is an urgent need to identify novel approaches to extrapolate acute in vitro results for the prediction of chronic in vivo effects. To this end, systematic mechanism-based nanotoxicity research based on in vitro high-throughput approaches and well-tailored nanostructures open new opportunities in toxicity prediction [17]. Particularly, understanding the individual effects of varying physicochemical parameters in well-controlled in vitro settings can help us understand the structural basis of nanotoxicity, which would allow for the development of safer nanomedicine by adopting a ‘safer-by-design’ approach. In turn, these strategies could enable the rapid transition of AuNPs from bench to bedside [17].

**Fig. 1:**
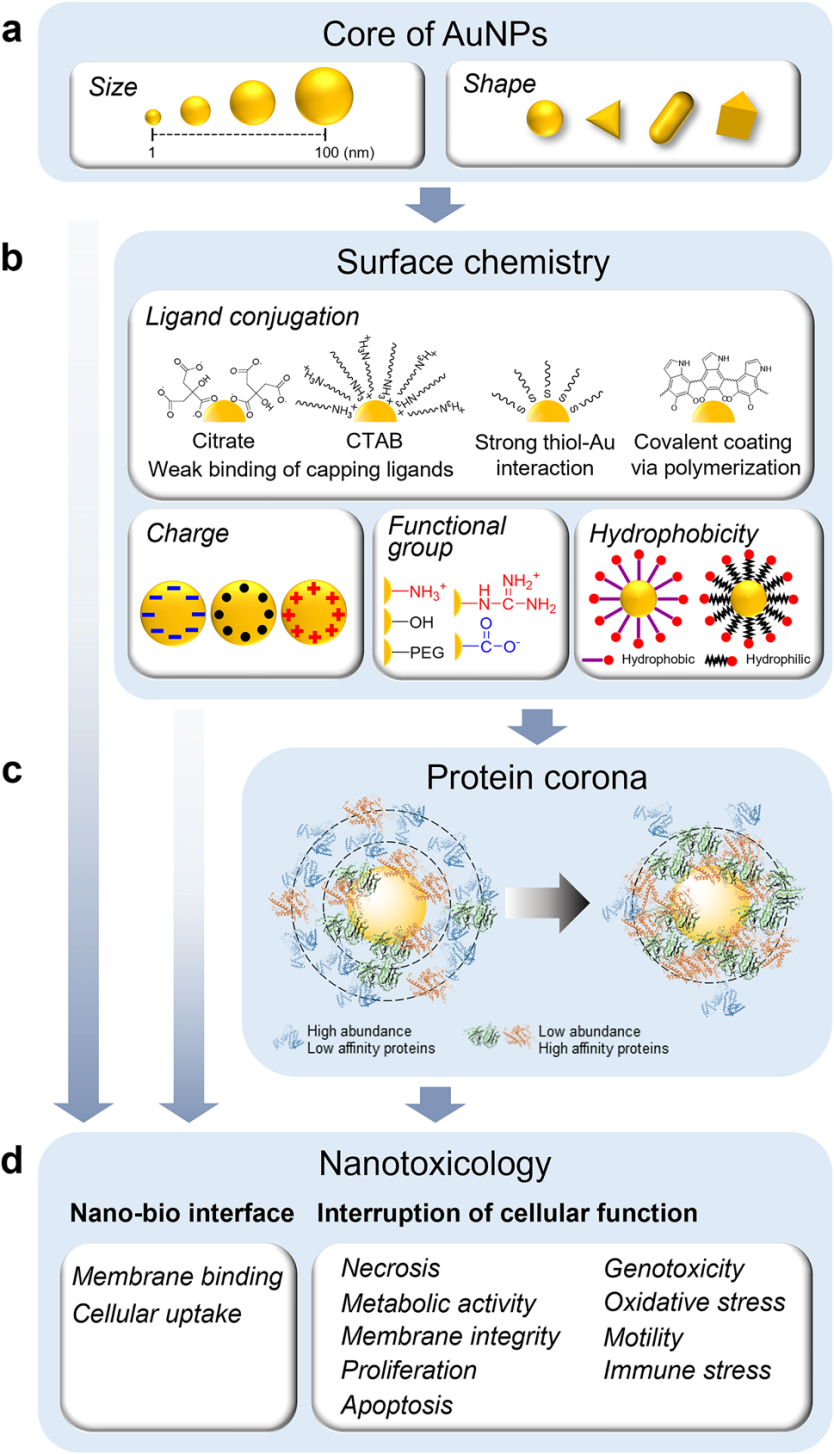
Physicochemical parameters of AuNPs assessed in nanotoxicity studies. **a** Core size and structure; **b** surface chemistry; **c** protein corona; **d** nanotoxicology

In this review, we first summarize in vitro nanotoxicology studies that have investigated the biological effects of AuNPs with different physicochemical parameters to elucidate how each variable determines their mechanisms of interaction. We will then discuss how the size and shape of the AuNPs alter their biological fate, as well as the effect of surface properties such as ligand conjugation mode, charge effect, functional groups, and ligand hydrophobicity, as well as protein corona formation (Fig. 1) [18]. We then briefly discuss well-known mechanisms of acute nanotoxicity such as AuNP-induced necrosis, apoptosis, and oxidative damage, as well as the analytical techniques required to assess these endpoints. Additionally, we reviewed the detailed mechanisms of AuNP-induced functional interference at NOAEL (no-observed-adverse-effect level) concentrations, including membrane rupture, cell motilities, immune responses, and genotoxicity, which can provide crucial information for the assessment of potential risks. Furthermore, we expect that this review will provide critical information for the development of nano-Quantitative Structure–Activity Relationship (QSAR) models, in addition to establishing a reference for safer AuNP design for biomedical applications, thereby opening up new possibilities for the development of more effective therapies in clinical settings.

## AuNPs with various physicochemical parameters and their biological interactions

The biological interactions of AuNPs are determined by their physicochemical properties such as their size, shape, surface charge, surface hydrophobicity/hydrophilicity, and surface functionalization. Understanding how each factor alters their biological effect, independently or in a combined manner, is crucial for the assessment of the risks associated with the clinical use of nanomedicine. In this section, we will review recent studies that evaluated how AuNPs with different core or surface properties affect biological systems differently.

### Biological effects of AuNP core size and shape

#### Core size of AuNPs and their biological interactions

The size of AuNPs is an important parameter when determining their biological properties. The core size affects endocytic processes, cellular localization, and targeting biomolecules, as NPs tend to interact with similar-sized biomolecules [19]. Many studies have assessed the effects of core size on the biological interactions of AuNPs, and nanotoxicity does not appear to be linearly correlated with core size. Concretely, a large proportion of the aforementioned studies suggest that smaller AuNPs are more toxic [20–23], whereas several others have reported the opposite [24].

The higher toxicity of small size AuNPs is associated with their greater cellular uptake efficiency [20–23], and the effective intracellular uptake of small-sized AuNPs is explained by the principle of particle wrapping [25, 26]. Receptor-mediated internalization accompanies the rearrangement of cellular receptors to locate around AuNPs, thereby generating sufficient free energy for particle wrapping. In this process, smaller particles require less energy than larger particles, which require a greater number of cellular receptors. Therefore, there may be a gradual depletion of receptors on the cell surface, thus limiting and slowing the internalization process [27]. On the other hand, other studies have proposed potential mechanisms through which larger AuNPs can exert a stronger toxic effect than smaller AuNPs. For example, Mironava et al. reported that 45 nm AuNPs exhibited significantly higher toxicity than 13 nm AuNPs and suggested that larger AuNPs sequestered inside the vacuole caused greater damage by disrupting the equilibrium of the inner surface of the vacuole. In turn, this leads to vacuole collapse and the release of AuNPs into the cytoplasm to disrupt normal cell functions [24, 28]. Furthermore, the specific interaction of NPs with similar-sized biostructures is another potential mechanism of toxicity. For example, DNA damage induced by the tight binding of AuNPs to the DNA major groove has been reported by several groups [20, 29]. Computer simulations conducted by Izanloo suggested that approximately 1.8-nm AuNPs can bind to the major groove of DNA and Pan et al. experimentally demonstrated that 1.4-nm AuNPs bind to the major groove of DNA with high selectivity and stability [29]. In their study, 1.4-nm AuNPs exerted toxic effects at concentrations 4–6 times lower than those of 1.2 and 1.8 nm AuNPs [20]. The authors hypothesized that this binding could block transcription in general, and this mechanism requires stringent size restrictions for steric reasons thus NPs larger or smaller than 1.4 nm are less likely to interact with DNA.

These examples suggest that AuNPs could exert cytotoxic effects, albeit through different mechanisms. The toxicity of small AuNPs has been attributed to a higher cell uptake, whereas that of large AuNPs has been linked to vacuole damage, and AuNPs of specific sizes induced toxic effects through interaction with biological structures of similar size. Therefore, the size of NPs is an important factor to consider when designing nanomedicine. For example, smaller AuNPs are considered good contrast agents due to their higher circulation time in the blood. Additionally, cellular uptake efficiency and drug loading efficiency must also be considered when designing drug delivery systems [30].

#### Core structure of AuNPs and their biological interactions

**Fig. 2:**
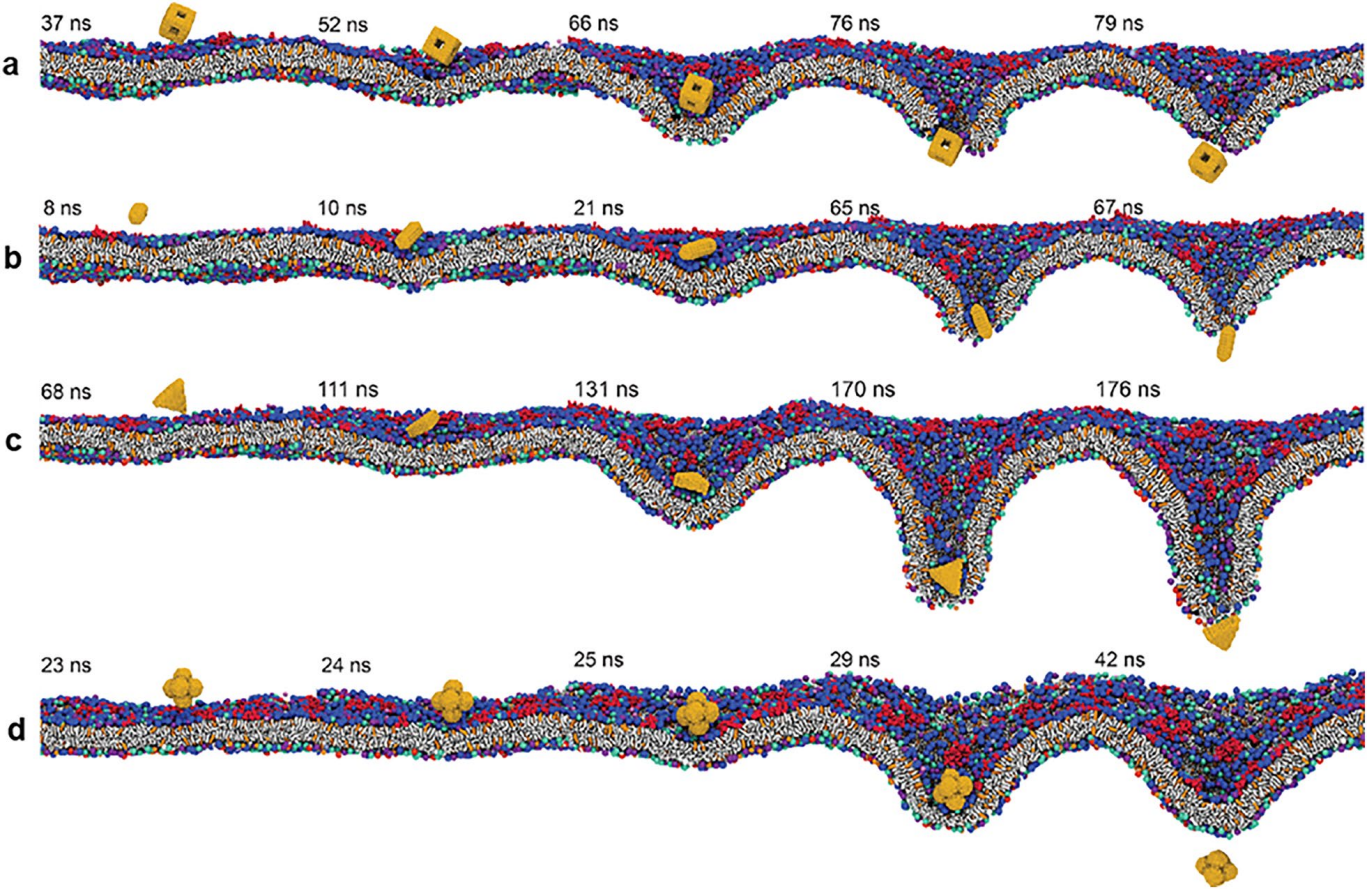
Snapshots of different shapes of AuNPs approaching the plasma membrane and undergoing reorientation during translocation. **a** Nanocage, **b** nanorod, **c** nanoplate, and **d** nanohexapod, each with the longest characteristic length of 2 nm. Water molecules both in the outer and inner cell are not shown for clarity. Reprinted with permission from [37], Copyright 2019, American Chemical Society

Gold nanospheres (AuNS) were first synthesized by Turkevich in 1951. Since then, several methods have been developed to synthesize AuNPs of various shapes, including nanospheres, nanotriangles, nanoprisms, nanorods, etc. Additionally, many report suggested unique optical properties (e.g., surface plasmon resonance) of anisotropic NPs make them of special interest for medical applications [31–33]. However, other studies have linked precisely these superior optical properties with nanotoxicity, and therefore understanding the mechanisms of shape-dependent nanotoxicity is critical for the successful implementation of anisotropic NPs in clinical applications. AuNPs with different shapes are often generated using different surfactants, and therefore the toxicity of the surfactant must also be taken into account in nanotoxicity studies. To isolate the effect caused by the core structure, other variables such as size and surface ligands must first be unified. Although some early studies reported increased toxicity with increased NP anisotropy [e.g., gold nanorods (AuNR)], later studies suggest that the reported cytotoxicity of anisotropic NPs is likely due to the toxic surfactant cetyltrimethylammonium bromide (CTAB), which is often used for the synthesis of AuNR [34]. For example, Arnida et al. compared the toxicity of PEGylated AuNSs and AuNRs and reported that although the cellular uptake of PEGylated AuNRs was more efficient compared to PEGylated AuNSs, neither PEGylated AuNSs nor AuNRs interfered with the proliferation of prostate cancer cells [35]. Zhang et al. compared the biological interactions of three different mPEG-coated anisotropic AuNPs, namely stars, rods, and triangles, and observed higher cellular uptake of gold nanotriangles (AuNTs) by macrophages, followed by AuNRs and gold nanostars [36]. Once again, these findings confirmed that there is a direct relationship between cell permeability and increases in anisotropy [37]. Molecular dynamics simulation analysis suggested that AuNPs with more vertices were more effective for cellular uptake compared to other shapes because AuNPs reoriented themselves and used sharp tips/edges to disrupt the membrane before entering the cell (Fig. 2). Similarly, Wang et al. reported that the shapes of gold nanoplates affected their cellular uptake efficiency and cytotoxicity. Specifically, the nanoplates with the sharpest angles (60°) were more readily uptaken and more cytotoxic than gold nano-pentagons and gold nano-hexagons [38].

In addition to cell permeability, other studies have also suggested that core structure affects the in vivo biodistribution of AuNPs. Arnida et al. compared the biodistribution of nanostars and AuNRs in ovarian tumor-bearing mice and found that AuNRs had a longer circulation time in the blood and preferentially accumulated in solid tumors compared to nanostars [39]. This result indicates that the core structure is a major parameter that determines cellular uptake efficiency as well as biodistribution. Therefore, this factor must be carefully considered when using AuNPs for nanomedicine, and more specifically when exploiting their anisotropic properties such as in photothermal or photodynamic therapy.

### Role of NP surface chemistry on nanotoxicity

The methods to introduce ligands onto the surface of AuNPs can be broadly divided into three categories: weak binding of capping ligands to AuNPs, strong thiol-gold interaction, and covalent coating via polymerization [40–42]. Thiol-containing and covalent coating-type ligands endow AuNPs with new lasting properties, whereas the capping ligands (e.g., citrate ions that electrostatically bind to AuNPs) transiently enhance the colloidal stability of NPs via weak interactions. Physically adhered capping ligands can be easily detached from the NP surface (6.7 kJ/mol) during cell treatment. Widely used thiol ligands strongly interact with the surface of gold (126–167 kJ/mol) but they can be progressively replaced by molecules with thiol groups in biological fluids [43]. Covalent coating via polymerization (259–345 kJ/mol) is less tailorable despite being more stable in biological environments. In early studies, nanotoxicity is often reported without distinction of the ligand conjugation method, and therefore the cytotoxicity caused by the capping ligand must also be independently considered. Here, we mainly discuss the role of surface chemistry on nanotoxicity based on studies carried out using thiol-ligands or covalently bound ligands to exclude the toxic effects caused by toxic surfactants such as CTAB, CTAC, and SDS [34, 44].

Relevant surface ligand parameters include surface charge and functional groups, as well as hydrophobicity. The surface charge of AuNPs is often considered a major factor associated with their nanotoxicity [45]. Many groups have suggested that the positive charge on the AuNPs' surface is the main cause of their toxicity, as these NPs can interact with negatively charged cell membranes. Additionally, many studies have experimentally demonstrated that cationic NPs are more toxic [46–48]. Nevertheless, many other reports have identified non-toxic cationic NPs, which contradicts the aforementioned findings. For example, Shukla et al. showed that lysine and poly(L-lysine) conjugated cationic AuNPs were not cytotoxic and that the levels of reactive oxygen species (ROS) inside the cells were reduced by lysine-AuNPs [49]. Furthermore, Schaeublin et al. argued that both positively and negatively charged AuNPs are cytotoxic, with negatively charged particles evoking stronger responses [50].

**Fig. 3:**
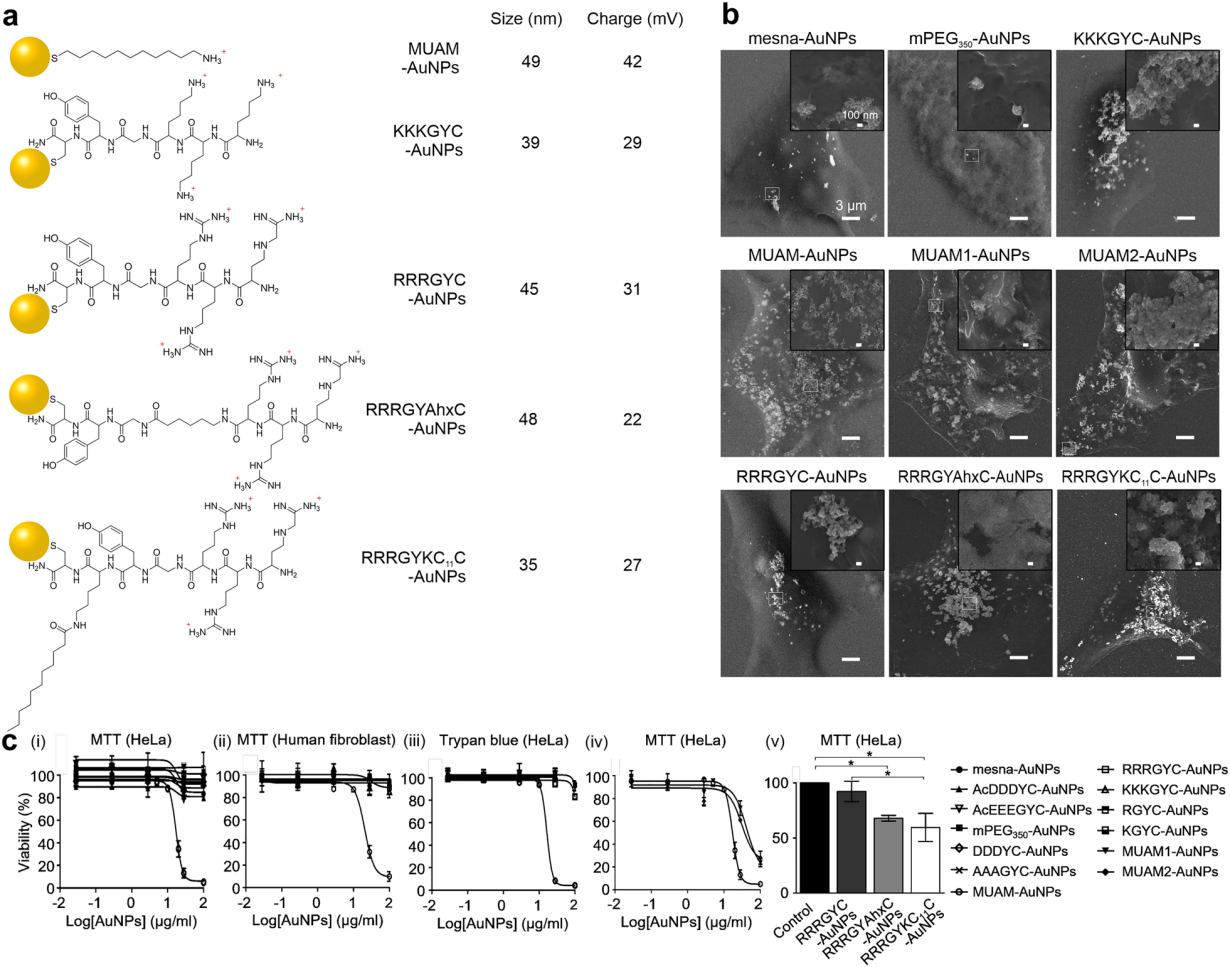
AuNPs modified with various cationic ligands and their biological effect. **a** Chemical structure and characterization of five different cationic AuNPs. **b** FE-SEM imaging of modified AuNP-treated HeLa cells. **c** Effect of modified gold nanoparticles (AuNPs) on cell viability (both MTT and Trypan blue assay)

To elucidate the effect of surface chemistry on nanotoxicity, many groups have conducted systematic studies using well-tailored systems to evaluate the individual and combined effects of surface parameters. Lee et al. analyzed the surface property-dependent nanotoxicity of a library of 15 different AuNPs with surface charges ranging from − 43 mV to  + 42 mV, in addition to varying functional groups and hydrophobicities [51]. Upon comparing three different cationic NPs, namely 11-amino-1-undecanethiol (MUAM,  ~ 42 mV), RRRGYC (~ 31 mV), and KKKGYC (~ 28 mV), only MUAM-terminated AuNPs was found to be cytotoxic, whereas all three cationic NPs showed increased interaction and higher cellular uptake efficiency (Fig. 3). In their work, neutral or anionic AuNPs were rarely found near the plasma membrane via FE-SEM imaging (Fig. 3b).

Given that these studies demonstrated that not all cationic AuNPs were cytotoxic, further analysis was carried out for three cationic AuNPs to elucidate the structural origin of observed cytotoxicity. First, the magnitude of positive charge was considered as a major cytotoxicity-determining factor, as the cytotoxic MUAM exhibited a higher surface charge. The strength of the surface charge of MUAM-AuNP was modulated by controlling the ligand density on the surface of the NPs to make MUAM1-AuNP (~ 34 mV) and MUAM2-AuNP (~ 30 mV) and these charged-reduced MUAM-AuNPs showed LD_50_ values similar to those of MUAM-AuNPs (LD_50_ of 17.1 µg/ml). This result strongly suggests that cytotoxicity is induced by factors other than the magnitude of the positive charge.

Next, the presence of primary amine groups was also considered as a major cause of cytotoxicity, as previous studies have associated the primary amine groups with toxicity [52, 53]. However, in Lee’s work, among two cationic AuNPs containing primary amine groups, modified with either MUAM or KKKGYC peptide ligands, only MUAM-AuNPs exhibited substantial cytotoxicity (Fig. 3c). These results suggest that primary amines are not the main cause of cytotoxicity.

**Fig. 4:**
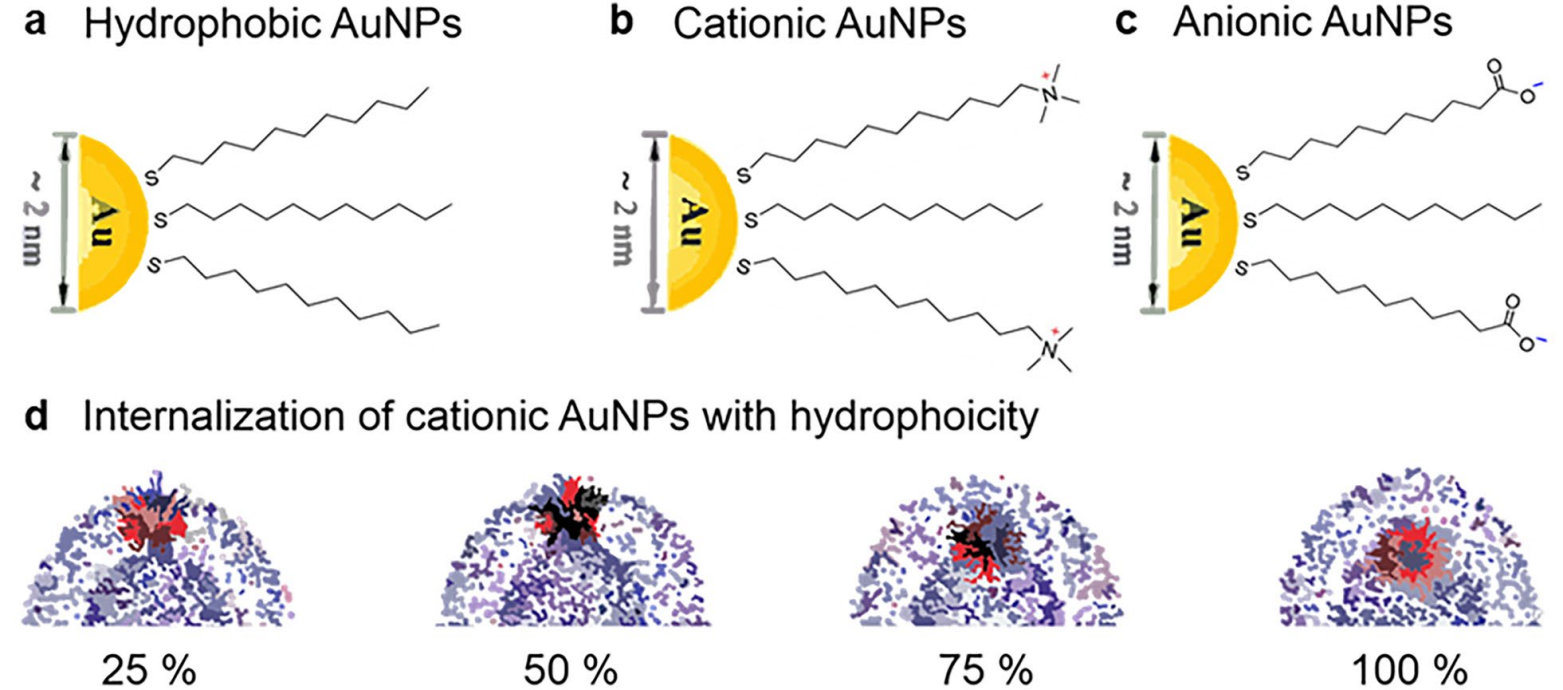
Schematic illustration AuNPs decorated with different surface ligands and their interaction with synthetic vesicles. Schematic diagram of AuNPs coated with **a** hydrophobic ligand, **b** cationic ligand, and **c** anionic ligand. **d** Simulation snapshot of the AuNPs coated with hydrophobic and cationic ligands entering the lipid membrane

Additionally, the effect of hydrophobic residues located near cations was considered to be the structural basis of the observed nanotoxicity, as several independent groups have suggested based on simulation studies and studies using model vesicles [54, 55]. For example, Quan et al. demonstrated that cationic AuNPs with a hydrophobic residue readily cross the vesicle membrane through a membrane pore and reach the vesicle core via strong electrostatic interactions, resulting in membrane rupture [55]. The hydrophobicity on the surface of AuNPs allows them to penetrate the outer layer of the lipid membrane and become stably embedded into the hydrophobic region of the vesicle, whereas their strong electrostatic interaction is attributed to their positive charge, resulting in membrane penetration (Fig. 4). Using model systems, Morillas-Becerril et al. also reported that the membrane perturbation activity of AuNPs is modulated by variations in their chemical structure, including the nature of the positive charge and the features of the underlying chains [54]. Positively charged trimethylammonium AuNPs with different hydrophobic chain lengths inducted varying effects: the long-chain ammonium-bearing AuNP induced a greater release of fluorescent dye from synthetic vesicles, whereas AuNPs with short-chain ammonium groups exerted a much weaker effect.

Lee et al. also focused on the hydrophobic residues around the positive charge [51]. They introduced hydrophobic moieties into the non-toxic cationic peptide ligand RRRGYC to produce RRRGY(Ahx)C and RRRGYK(C_11_)C (Fig. 3a). Using FE-SEM analysis and MTT assays, the authors demonstrated that the introduced hydrophobic moieties enhanced the cellular uptake of AuNPs and increased cell death, respectively (Fig. 3b and c). Collectively, these results suggest that the hydrophobic interaction provided by the alkyl chain of the inner portion of the ligand shell plays an important role in the ability of NPs to rupture membranes and penetrate cells, resulting in cell death.

### Effect of protein corona

**Fig. 5:**
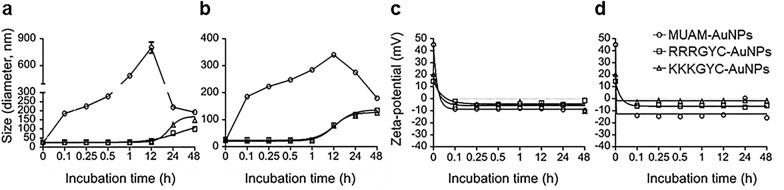
Time-dependent study on the protein corona formation of cationic AuNPs in biological media. The sizes of AuNPs were measured using DLS in DMEM containing 1% FBS (D1) (**a**) and DMEM containing 10% FBS (D10) (**b**). The surface charge of cationic AuNPs was measured in D1 (**c**) and D10 (**d**) media

In biomedical applications of AuNPs, in vivo injection and subsequent exposure to the biological environment are natural. The surface of NPs is immediately (i.e., within the first few minutes) covered with various proteins that form the so-called “protein corona” (PC) via electrostatic, hydrophobic, and van der Waals forces [56, 57]. The PC is composed of both a hard and soft corona, where the hard corona contains proteins that bind tightly to NPs, whereas the soft corona has proteins that bind loosely [58, 59]. The formation of the PC is a dynamic and competitive process, in which the early formed soft corona is progressively replaced by a hard corona (Fig. 5a) [51, 60–63]. This exchange process is important when particles redistribute upon uptake into cells from the bloodstream or upon transport from one organ to another [64].

Many reports have suggested that PC formation reduced nanotoxicity, as the PC increases the size of AuNPs and shifts the surface charge to negative values, thus reducing the direct contact of AuNPs with the cell membrane. In turn, this leads to a concomitant decrease in the uptake efficiency [65, 66]. For example, Cheng et al. demonstrated a significant decrease in cellular uptake efficiency of AuNPs in Dulbecco Modified Eagle Medium (DMEM) supplemented with 10% FBS compared to serum-free DMEM [67, 68]. Choi et al. also reported that the cytotoxicity induced by positively-charged AuNPs decreased in human plasma and disappeared completely after PC formation in HSA [69]. On the other hand, increased toxicity due to PC formation has also been reported. Mazzolini et al. suggested that the adsorbed proteins can interact with specific receptors on the cell surface, resulting in an increase in cellular internalization via the receptor-mediated endocytosis mechanism. For example, the authors demonstrated that transferrin (Tf) from PC could induce NP internalization via interaction with the Tf receptor (TfR) [70]. Digiacomo et al. also suggested that apolipoproteins, which are the main components of the PC, may play an important role in triggering the receptor-mediated uptake of NP into cells [71]. Alternatively, Deng et al. suggested that proteins adsorbed to the NPs surface undergo structural denaturation, which exposes epitopes to the surface, potentially resulting in increased immunogenicity. Enhanced immunogenicity may increase the risk of cytoplasmic and systemic toxicity by increasing internalization specificity, especially in professional and nonprofessional phagocytes such as macrophages and endothelial cells. For example, the presence of poly(acrylic) acid on the surface of AuNPs has been found to induce the unfolding of adsorbed fibrinogen that, in turn, interacted with the leukocyte receptor MAC-1, thereby triggering an inflammatory response [72–74].

PC encapsulating NPs inevitably affect the surface properties of NPs. Therefore, the use of AuNPs as nanomedicine materials not only requires a systematic evaluation of their nanotoxicity but also their functional changes upon corona formation. First, the PC coated on the NPs surface affects the targeting ability of the NPs, and this effect tends to depend on the size of the targeting moiety. Smaller targeting moieties experience a greater shielding effect, whereas the opposite occurs in larger targeting moieties. For example, Varnamkhasti et al. showed that NPs functionalized with relatively small targeting moieties (e.g., aptamers) lost specificity after PC formation [75]. In contrast, Dai et al. confirmed that NPs functionalized with antibodies, which are relatively large targeting moieties, retain their targeting ability after PC formation [76]. Furthermore, Xiao et al. compared the targeting ability of AuNPs functionalized with targeting moieties of different sizes, namely RGD and Tf, before and after PC formation [77]. Prior to PC formation, RGD-AuNPs initially exhibited higher targeting ability than Tf-AuNPs. However, this trend was inverted after PC formation, suggesting that the masking effect of PC is more predominant on smaller targeting moieties. While these studies suggest that PC significantly alters the biological behavior of NPs, including internalization, toxicity, and targeting ability, the surface properties of the NPs were still partially maintained. Interestingly, positively charged NPs interact better with cells than their negatively charged counterparts after PC formation even though similar zeta potential values were observed after corona formation regardless of the initial charge (Fig. 5b) [78].

## In vitro nanotoxicity assay

Nanomaterials can interact with biological systems in various ways, and therefore several approaches have been utilized to conduct risk assessments both in vivo and in vitro. This review will focus on in vitro cytotoxicity assays, as they not only provide an ethical and cost-effective means to monitor NP toxicity but also allow for the detailed characterization of the mechanisms by which NPs affect biological systems (Table 1) [79]. These mechanistic studies will provide important information for the prediction of NP toxicity. NPs can directly affect cell viability, resulting in necrosis or apoptosis at high concentrations. However, they can also interfere with many cellular functions at low concentrations resulting in chronic toxicity or a disruption of the homeostasis of biological systems. Direct effects on viability are often assessed by monitoring metabolic activity, membrane integrity, proliferation, and apoptosis. On the other hand, indirect toxicity can be assessed by monitoring motility, genotoxicity, and oxidative stress. A multifaceted analysis of the cellular effects of NPs is necessary when assessing the biological effects of NPs, and analytical methods should be carefully selected and designed based on the expected toxicity mechanisms of the nanomaterials. Here, we summarized representative assay methods according to the cell function to be observed.

**Table 1:** Analytical methods for in vitro assessment of nanotoxicity

Toxic effect	Measurement	Assessment tools	Refs.
Metabolic activity	Mitochondrial activity assay	MTT assay	[80–83]
	Esterase activity	LIVE/DEAD assay	[84]
Membrane integrity	Membrane damage	LDH assay	[79]
		Trypan blue	[79]
		Propidium iodide	[79]
		Patch-clamp experiment	[85]
	Lysosomal activity	Neutral red	[80]
		Lucifer yellow	[86]
Apoptosis	Apoptotic signal	Annexin V	[87]
		Caspase-3	[80, 87]
Proliferation	Colony forming efficiency	Cologenic/clonogenic assay	[88]
Genotoxicity	DNA damage	Comet assay	[89, 90]
		Micronucleus assay	[91]
		TUNEL assay	[92]
	Gene mutation	Ames test	[92]
	Gene expression	qPCR	[92]
		Microarrays	[92]
	DNA replication	BrdU assay	[93]
Oxidative stress	Reactive oxygen species (ROS)	DCFH	[90, 94]
		EPR	[91, 94–97]
	Lipid peroxidation	TBA assay for MDA	[91, 94, 98]
		Amplex red	[94]
Immune stress	Cytokine	ELISA	[90, 99, 100]
		Western blotting	[101]
Motility	Cell migration	Wound healing assay	[102]
		Invasion assay	[51]
	Cytoskeletal structure	Phalloidin staining	[51]

### Viability assays

Cell viability can be assessed by monitoring metabolic activities, membrane integrity, cell proliferation, and apoptotic signals. Particularly, mitochondrial activity and esterase activity assays are among the most common approaches to analyze metabolic activity. Mitochondrial activities are measured using tetrazolium dye [3-(4,5-dimethylthiazol-2-yl)-2,5-diphenyltetrazolium bromide (MTT)]. The colorimetric MTT assay is based on the reduction of the yellow tetrazolium dye to a purple water-insoluble formazan precipitate in cells with intact mitochondria [80]. Simple spectroscopic monitoring of formazan formation provides quantitative information on the ratio of metabolically active cells. However, a careful interpretation of the data is needed as the mitochondrial activity result is often uncoupled with other viability assays such as proliferation or membrane integrities [79, 81–83]. There are various alternatives of tetrazolium dyes available for analogous assays such as XTT or WST1, which produce soluble dyes. When selecting these colorimetric assays, it is also important to consider the absorption wavelength of AuNPs. The monitoring of esterase activity using calcein acetoxymethyl ester is another option for the assessment of metabolic activity. Calcein acetoxymethyl ester undergoes enzymatic hydrolysis by active esterases within living cells, which results in a green-fluorescent product. This assay is often used in combination with the ethidium homodimer assay, which labels necrotic cells with red fluorescence by targeting the DNA of damaged cells [84].

Compromised membrane integrity is a key characteristic of necrotic cells. The integrity of the plasma membrane can be assessed by: (1) vital staining using dyes such as Trypan Blue (TB) or propidium iodide (PI), (2) monitoring the leakage of active enzymes into the culture media, and (3) electrophysiological techniques, such as voltage clamp, using microelectrodes, which directly measure ionic currents through the cell membrane [79, 85]. The former two methods are more adequate for toxicity studies, as they allow for the simultaneous screening of multiple cells to determine the average toxic effect on the ensemble. For vital staining, the ratio of cells stained with the charged dyes (TB or PI) is determined. In a typical TB assay, TB dye is excluded from live cells with intact membranes, whereas cells with damaged membranes allow for the passage of TB into the cytosol where it shows a strong absorption at 605 nm. PI intercalates between the bases of DNA and dsRNA and emits a fluorescent signal at 617 nm when entering the cells [79]. Alternatively, weakly ionic neutral red (NR) dyes are used to monitor the integrity of the lysosomal membrane. NR is uptaken into the cytosol by non-ionic diffusion through the cell membrane, after which it accumulates in the lysosomes of viable cells [80]. Severe damage to lysosomal membranes leads to the release of the fluorescent dye Lucifer yellow [86].

Apoptosis is a form of programmed cell death that has been extensively studied in nanotoxicological research. This endpoint is often assessed by monitoring apoptotic signals, such as plasma membrane inversion or Caspase-3 signaling. Upon the onset of apoptosis, the inner and outer sides of the plasma membrane become inverted, resulting in the exposure of intracellular phosphatidylserine to the extracellular space. Fluorescently labeled Annexin V is regularly used to detect apoptotic cells, as it binds to phosphatidylserine exposed on the surface of apoptotic cells. The activation of Caspase-3 activity is another distinctive sign of apoptosis. Specifically, Caspase-3 activation triggers multiple apoptotic signaling cascades, after which the procaspase-3 zymogen is produced and activated by upstream signals [87]. Activated Caspase-3 is known as an “executioner” caspase because cell death is inevitable once it is activated. Caspase-3 activity is often monitored by cleaving DEVD, a Caspase-3 specific substrate, linked to a chromophore (p-nitroanline) or a fluorophore (7-Amino-4-trifluoromethylcoumarin, 7-amido-4-methylcoumarin), which absorbs or emits light when separated from the substrate, respectively [80].

Upon treatment with NPs, some cells lose their proliferation capability despite not being necrotic or apoptotic. To quantify this, cell proliferation assays are performed by counting colonies of highly proliferating cells by visual inspection after exposure to nanomaterials [88]. Clonogenic assays allow for the characterization of the effects of specific agents on the survival and proliferation of cells. One of the advantages of this method is that the cells are not exposed to any other agents then nanomaterials excluding the side effect. Each colony is then stained with crystal violet or nuclear stains for counting by visual inspection.

### Functional assays

#### Genotoxicity

NP-induced genotoxicity involves DNA damage, DNA mutation, impaired DNA replication, and changes in gene expression profiles. DNA damage often results in DNA fragmentation, which can be seen through the comet assay, micronucleus assay, and TUNEL assay. The comet assay, also known as single-cell gel electrophoresis, provides a sensitive and rapid means for quantifying and analyzing DNA damage in individual cells [89, 90]. Each cell is embedded in agarose gel and the mobility of DNA under an electric potential is monitored after cell lysis and removal of cellular proteins. Broken fragments of DNA and damaged DNA migrate away from the nucleus and are visualized using a DNA-specific fluorescent dye. The micronucleus assay is based on the microscopic detection of a chromosome or chromosome fragment from a cell, which has failed to integrate into the nucleus of its daughter cell after division. This assay provides information not only on background micronuclei levels but also cell proliferation by distinguishing mononuclear from binucleated cells [91]. The TUNEL assay labels the ends of DNA with biotinylated dUTP, which can be optically detected via light microscopy using streptavidin–horseradish peroxidase and a diaminobenzidine chromogen. Alternatively, the fluorescently labeled dUTP nucleotides can be visualized using fluorescent microscopy [92].

Nanomaterial-induced DNA mutation can be characterized using the Ames test, a common mutagen screening assay. In this reverse mutation assay, histidine negative *Salmonella typhimurium* cells, which are unable to grow in the absence of histidine, are exposed to NPs. Reversion to a histidine-positive phenotype (indicating a reverse mutation in the histidine locus) after NP treatment suggests that the NPs possess mutagenic properties. NPs can also indirectly affect genomic functions by altering gene expression profiles resulting in various phenotypic changes [92]. The levels of gene expression can be monitored by analyzing the amount of target mRNA using reverse transcriptase PCR or microarrays [92]. Additionally, inhibition of DNA replication by AuNPs has also been determined using the bromodeoxyuridine (BrdU) incorporation assay. The BrdU assay has long been used to detect DNA replication, where BrdU is a thymidine analog that differs from thymidine in its substitution of bromine for a methyl group. BrdU competes with thymidine for incorporation into newly synthesized nuclear DNA during the S-phase of the cell cycle. Incorporation of BrdU is visualized using immunostaining following DNA denaturation [93].

#### Oxidative stress

Nanomaterials may also disturb the oxidative balance of the cell. This phenomenon is known as oxidative stress and it results in increased concentrations of intracellular ROS. The generation of abnormally high concentrations of ROS can have many toxicological implications leading to abnormal cellular function [79]. Oxidative stress can be monitored via direct measurement of ROS level or by indirect assessment of oxidative damage. ROS levels can be directly measured using a ROS reacting dye such as 2′,7′-dichlorodihydrofluorescein (DCFH) or through electron paramagnetic resonance (EPR) measurements [94]. In the DCFH assay, the diacetate precursor of DCFH is hydrolyzed by esterases generating the non-fluorescent DCFH, which can then be converted to a fluorescent product by ROS [90]. EPR is also a popular technique that has been widely used to assess NPs and particle-induced ROS generation [91, 95]. The use of specific spin traps based on nitrones or nitroso compounds such as 5,5-dimethylpyrroline-N-oxide (DMPO), α-phenyl-tert-butylnitrone (PBN), and 2-methyl-2-nitrosopropane (MNP) allows for the quantification and specific identification of the generated free radical species, whereas this level of specificity is not possible with the DCFH assay [91, 96, 97]. On the other hand, oxidative stress can be estimated by monitoring the formation of oxidation products of cellular components (e.g., lipid peroxide, an oxidation product of polyunsaturated fatty acid) using TBA or Amplex red [94]. Lipid peroxides, which are produced due to the oxidation of fatty acids with three or more double bonds, are unstable and decompose to form a complex series of compounds, which include reactive carbonyl compounds [e.g., malondialdehyde (MDA)] [98]. The generated MDA reacts with thiobarbituric acid (TBA) forming a pink chromogen (TBARS), which is measured at 532–535 nm [91].

#### Immune stress

Exposure to NPs can induce unnatural immune responses by immune cells. Immune responses often result in increased production of inflammatory mediators, chemokines, immunoglobulin isotypes, intracellular signaling molecules, apoptotic mediators, adhesion molecules, and antibodies [99]. Representative markers of immune responses include IL-1β, IL-6, IL-8, cathepsin L, cathepsin B, and TNF-α, which can be quantitatively measured via routine immunoassays such as ELISA or western blotting [90, 100, 101].

#### Cell motility

Cell motility is a critical cellular function, as it is a crucial process for the survival and differentiation of mammalian cells. This endpoint can be monitored by gap-filling (wound healing) assays or invasion assays, which are standard in vitro techniques for probing collective cell migration in two or three dimensions, respectively. In these assays, the migration behaviors of cells from a confluent monolayer to a cell-free area are monitored by visual inspection [102]. Monitoring of cytoskeletal structure is often carried out in conjunction with motility assays to elucidate the causes of impaired motility. The cytoskeletal structure is visualized using a fluorophore-phalloidin conjugate, which is a highly selective bicyclic peptide that binds to actin filaments (also known as F-actin). Cells lacking well-organized cytoskeletal structures exhibit disassembled and fragmented F-actins, whereas undamaged cells maintain elastic and elongated F-actin fibers. The loss of long F-actins could explain motility impairments because F-actins align with the migration axis to facilitate cell movement [51].

## Mechanisms of AuNPs toxicity

**Fig. 6:**
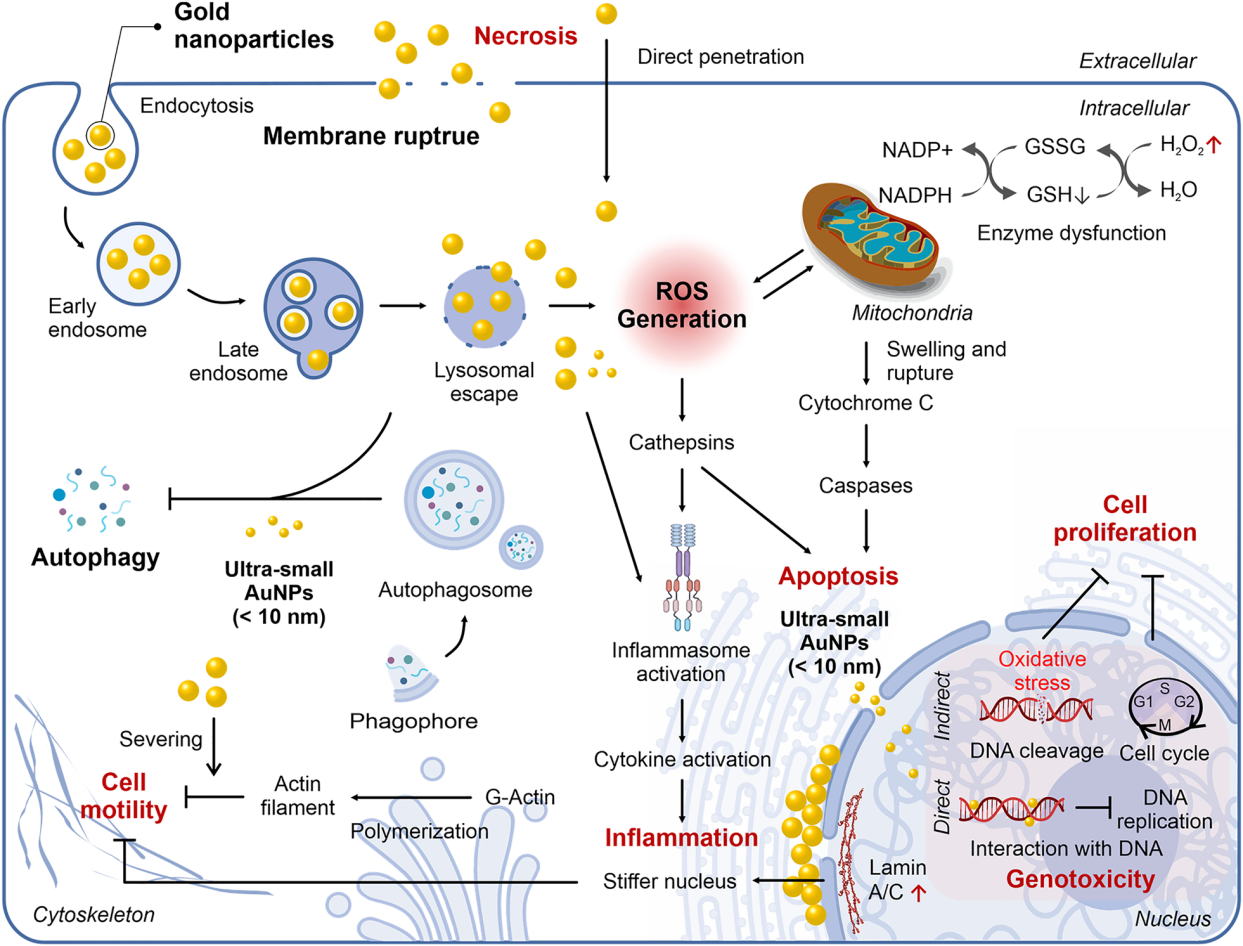
Potential mechanism of AuNP-induced toxicity. (i) Necrosis: direct physical damage to the cellular membrane, (ii) apoptosis: oxidative stress-triggered intrinsic mitochondrial-mediated pathway, (iii) inhibition of cell motility: interference of actin filament formation and stimulation of lamin A/C overexpression inducing increased nuclear stiffness, (iv) ROS generation: indirectly induced by AuNPs mainly through lysosomal membrane permeabilization, mitochondrial depolarization, and interaction with redox-active enzymes, (v) genotoxicity: primary damage induced by AuNPs including direct interaction with DNA leading to inhibition of DNA replication and indirect DNA cleavage and mutation via oxidative stress, and (vi) inflammation: induction of inflammatory response due to direct and indirect inflammasome activation by AuNPs

An in-depth mechanistic understanding of the effects of NPs on cellular functions is important when studying nanotoxicity. Different types of cell deaths, such as necrosis and apoptosis, can be triggered by NPs at high concentrations. However, the effects of NPs at sublethal concentrations still remains to be further elucidated. Understanding the secondary or long-term toxicity of NPs would allow for proper risk assessment and risk management to address concerns regarding the clinical application of NPs (Fig. 6). In this section, we will discuss the mechanisms through which different AuNP physicochemical parameters induce cell death, as well as functional changes to target cells.

### NP-induced necrosis via membrane disruption

Necrosis is a form of cell injury that results in premature cell death. Many studies have reported that the loss of structural integrity of the plasma membrane is a hallmark of necrosis and represents the common final point at which the cell can no longer maintain its discrete identity from the environment. As membrane disruption results in an uncontrollable exchange of intracellular and extracellular materials, necrosis can be assessed by monitoring the release of cytosolic enzymes (e.g., lactate dehydrogenase) or uptake of membrane-impermeable dyes as described in previous studies [48, 51, 55, 103]. The destructive effects of AuNPs on cell membranes are largely attributed to cationic functional groups with hydrophobic moieties in their structure. Specifically, the positive charges of the cationic groups interact strongly with the negatively charged membrane, whereas the hydrophobic moieties allow the AuNPs to become embedded in the hydrophobic tails of the lipid bilayer, resulting in membrane rupture [55]. Molecular dynamics simulations suggest that increasing the surface charge up to 50% beyond the optimum enhanced membrane penetrations, whereas further increasing the charge densities resulted in membrane disruption [48]. On the other hand, AuNPs modified with anionic functional groups neighboring hydrophobic moieties or with cationic functional groups without neighboring hydrophobic moieties directly penetrated the cells without causing apparent membrane perforation, albeit with different efficiencies [104]. These results provide a theoretical basis for the design of non-toxic nanocarriers that can cross cell membranes without destroying them.

Another necrotic mechanism induced by AuNPs is the disruption of the lysosomal membrane, which leads to the release of proteolytic enzymes into the cell, including proteases, RNAases, DNAases, and phosphatases [105]. When activated in the cytoplasm, these enzymes lead to DNA, RNA, and protein damage, resulting in cell death [106]. AuNP-mediated disruption of the lysosomal membrane is primarily related to the lysosomal proton sponge effect, in which cationic AuNPs in the low-pH lysosome induce an influx of Cl^−^ followed by an H^+^ influx, thereby altering the osmotic environment in the lysosome. Alternatively, AuNPs can physically interact with the lipid layer of the lysosomal membrane to induce membrane rupture. Some AuNPs have been found to induce necrosis by triggering the dispersal of a protease (cathepsin B) through the disruption of the lysosomal membrane [103]. Cathepsin B is considered the key inducer of cellular necrosis and the release of this protease leads to the activation of receptor interacting protein kinase-1 (RIPK1). In turn, RIPK1 interacts with RIPK3 to form a necrosome complex, which ultimately induces cell death [107].

### NP-induced apoptosis

The activation of apoptosis signal transduction pathways includes extrinsic and intrinsic pathways. Extrinsic pathways are mediated by death receptor superfamily proteins, such as CD95 and tumor necrosis factor receptor I, which are activated when death signals are received, resulting in cell apoptosis [108]. AuNPs-mediated apoptosis occurs via intrinsic mitochondrial-mediated pathways, in which caspase 3 activation plays a key role. Mainly, AuNP-induced oxidative stress leads to apoptosis through the following steps: (1) opening of a mitochondrial permeability transition pore (MPTP), (2) loss of mitochondrial transmembrane potential, and (3) release of pro-apoptotic proteins (e.g., Cyt c) from the intermembrane space into the cytosol [109]. AuNP-induced oxidative stress in mitochondria is triggered by ROS generation accompanied by lysosomal escape of AuNPs or by GSH depletion via AuNP-induced thiolate formation.

### Cell motility

Cell migration is a crucial process for the differentiation and survival of mammalian cells and several diseases have been linked to the abnormal regulation of cell migration [110]. Cell migration is controlled by various external signals and several studies have reported that AuNPs have an inhibitory effect on cell motility. In this context, two distinct inhibition mechanisms have been proposed: (1) AuNPs interfere with actin filament formation or (2) AuNPs induce nuclear stiffness, thereby slowing down cell migration [51, 111].

Several studies have reported AuNP-induced inhibition of cell migration accompanying the fragmentation of F-actin. The fragmented actin filament cannot consist of a dense F-actin meshwork at the cell edge. This impairs the generation of crucial cytoskeletal structures such as the lamellipodia, resulting in the inhibition of cell migration. Further studies were conducted to characterize the mechanisms that lead to F-actin fragmentation. Actin polymerization in the presence of AuNPs was monitored in vitro to investigate whether AuNPs interfere with actin polymerization directly or indirectly*.* Although the rate of actin polymerization was comparable in the samples with or without AuNPs, the actin filaments formed in the presence of cationic AuNPs were shorter and more nucleated compared with the untreated control. These results indicate that rather than inhibiting polymerization or altering related signaling pathways, the AuNPs used in this experiment directly severed the actin filaments resulting in fragmented and nucleated F-actins.

Other studies have indicated that AuNPs could significantly reduce cell migration by enhancing nuclear stiffness [111]. For example, a recent study reported that nuclear-targeting AuNPs with nuclear localization signal peptides (NH_2_-CGGGPKKKRKVGG-CO_2_H) were located predominantly on the nuclear membranes, thus providing mechanical support (i.e., increasing stiffness). However, AuNPs were rarely observed inside the nucleus, which was likely due to the large sizes of the NPs and their aggregates compared to the nuclear pores (~ 9–12 nm). The authors also proposed that the decrease in cell motility could also be due to an increase in lamin A/C expression. Specifically, the authors reported a strong upregulation of the lamin A/C protein, which is located in the inner nuclear membrane and functions as a structural component of the nuclear lamina to enhance nuclear stiffness. The mechanisms through which AuNP induces lamin A/C expression are not yet clearly elucidated. However, it has been suggested that this response constitutes a cellular defense mechanism to maintain the structural integrity of the nucleus in response to increased nuclear stiffness induced by the physical contribution of AuNPs.

### Oxidative damage and ROS generation

AuNP-induced ROS generation appeared to precede many other cellular responses, for example, ROS is generated in the lysosome, resulting in lysosomal dysfunction and lysosomal membrane permeabilization. In turn, this leads to the release of AuNPs and lysosomal components such as cathepsin to the cytoplasm [112]. The released AuNPs and disseminated cathepsin then induce mitochondrial depolarization and mitochondrial permeabilization, providing another route for ROS generation. Notably, some studies suggest that there is a strict permeability size limit (< 6 nm) by which AuNPs can pass through ion channels of the outer mitochondrial membrane, indicating that the disruption of the mitochondrial membrane by AuNPs is primarily mediated by dissolved metal ions that are small enough to move across mitochondrial pores [113]. Other major consequences induced by excessive cellular ROS production due to AuNP exposure include genetic damage and inflammation, as well as cell death via apoptosis, necrosis, and autophagy.

### Genotoxicity

There are several direct and indirect mechanisms through which nanomaterials could potentially cause DNA damage. Researchers have classified the mechanisms of genotoxicity as primary and secondary. Primary genotoxicity can be defined as genetic damage exerted by particles in the absence of inflammation. This primary genotoxicity implies both a direct physical interaction between NPs and the genomic DNA and indirect primary genotoxicity induced by the formation of ROS in NPs-activated target cells. Secondary genotoxicity refers to genetic damage that results from the oxidative DNA attack by ROS/RNS and possibly other mediators that are generated during particle-elicited inflammation from recruited and activated phagocytes, namely, macrophages and neutrophils [114]. The most obvious primary genotoxicity effects induced by AuNPs include DNA cleavage and mutation via oxidative stress, which are often investigated using the comet assay, Ames test, as well as TUNEL assay. Lee et al. earlier suggested indirect primary genotoxicity by demonstrating the inhibition of DNA replication as well as alteration of gene expression profiles when cells were exposed to cationic and hydrophobic AuNPs. Inhibition of DNA replication was monitored via the BrdU incorporation assay and DNA damage resulted in the complete inhibition of cell proliferation, which was also observed at the NOAEL concentration. Using gene expression profiling, this study also demonstrated a repression of the G1-to-S phase transition during the cell cycle [51]. Alternatively, ultra-small AuNPs can induce primary direct genotoxicity by strongly interacting with DNA within the cell nucleus to induce DNA conformational changes or inhibit DNA transcription [29, 115]. The presence of AuNPs near DNA induces the release of water molecules from the DNA duplex and the sodium cations appeared to be closely linked to the DNA. By conducting molecular dynamics simulations, Izanloo demonstrated that when  ~ 1.8 nm AuNPs come close to the DNA, the phosphate group directed the particles into the major grooves of the DNA molecule, thereby destabilizing the DNA structure [29]. Peeling and untwisting states were also observed at the DNA ends and the nucleotide base rested flat on the surface of the AuNP, thereby increasing entropy. Furthermore, the changes in conformational energy and the hydrogen bond numbers indicated that DNA becomes unstable in the vicinity of AuNPs. Additionally, Goodman et al. reported that small AuNPs (~ 2 nm) affect DNA transcription by causing structural changes in the DNA structure, thereby interfering with RNA polymerase activity [115]. The authors suggested that the tight binding of AuNPs inhibits the access of RNA polymerase to the double helix.

### AuNP-mediated immune responses

AuNPs could trigger inflammatory responses and could therefore potentiate innate immune responses and serve as an efficient immunotherapy. In a report by Zhu et al. small-sized (< 10 nm) AuNPs preferentially activated the NLRP3 inflammasome for Caspase-1 maturation and interleukin-1β (IL-1β) production, whereas larger AuNPs (> 10 nm) triggered the NF-κB signaling pathway [116]. The latter has been reported to be up-regulated through the expression of cathepsin, which is associated with the inflammatory response, by directly interacting with the AuNPs [100]. Ultrasmall (< 4.5 nm) AuNPs activate the NLRP3 inflammasome by directly penetrating the cell cytoplasm and targeting autophagy protein-LC3 for proteasomal degradation. These AuNPs promote the degradation of LC3, thus relieving the LC3-mediated inhibition of the NLRP3 inflammasome. The NLRP3 inflammasome and the release of IL-1β reportedly enhance the adaptive response against specific pathogens or malignancies. This result demonstrates that ultrasmall AuNPs can serve as adjuvants to enhance antigen-specific antibody production.

## Conclusions and future perspectives

Nanomedicine represents a new and promising means to treat diseases. However, the clinical application of nanotechnology remains limited mainly due to the concerns on uncertainties associated with the potential side effects of nanomaterials. Even though many reports suggest that AuNPs do not seem to exert toxic effects at the concentrations used in medical applications, there are still lasting concerns regarding the potential side effects of nanomaterials owing precisely to their size and unique physicochemical properties compared to their respective bulk materials. Additionally, extrapolating the results of in vivo acute toxicology tests to estimate the long-term cumulative effect of these materials remains an important challenge, which makes it difficult to assess their potential human health effects. In this context, in vitro mechanistic nanotoxicity studies carried out with careful manipulation of variables may provide a solid basis for predictive toxicology, which would promote the bench-to-bedside transition of nanotechnology-based therapies. Particularly, understanding the mechanisms of AuNP-induced cytotoxicity in relation to the physicochemical parameters of AuNPs is essential for designing low toxicity nanomaterials for risk management. In this review, the effects of various physicochemical properties known to be closely related to AuNP-mediated cytotoxicity such as size, shape, surface charge, functional groups, and hydrophobicity on cell viability and cellular function were carefully discussed with an emphasis on our current mechanistic understanding of nanotoxicology. We believe that this review provides critical information for the development of predictive nanotoxicology as well as for nano-QSAR models. This, in turn, establishes a theoretical framework for the design of safer AuNPs for biomedical applications. We also expect that our approach will contribute to the transition of nanotechnology from bench to bedside, thereby opening new possibilities for improved nanomedicine.

## Data Availability

Not applicable.

## Abbreviations

AuNPs: Gold nanoparticles
NPs: Nanoparticles
NOAEL: No-observed-adverse-effect level
QSAR: Quantitative Structure–Activity Relationship
AuNS: Gold nanosphere
CTAB: Cetyltrimethylammonium bromide
CTAC: Cetyltrimethylammonium chloride
SDS: Sodium dodecyl sulfate
AuNRs: Gold nanorod
AuNTs: Gold nanotriangles
PC: Protein corona
Tf: Transferrin
TfR: Transferrin receptor
MTT: 3-(4,5-Dimethylthiazol-2-yl)-2,5-diphenyltetrazolium bromide
TB: Trypan Blue
PI: Propidium iodide
NR: Neutral red
BrdU: Bromodeoxyuridine
ROS: Reactive oxygen species
DCFH: 2′,7′-Dichlorodihydrofluorescein
EPR: Electron paramagnetic resonance
MDA: Malondialdehyde
TBA: Thiobarbituric acid
RIPK: Receptor interacting protein kinase
Cyt c: Cytochrome c
CASP: Caspase
MPTP: Mitochondrial permeability transition pore
IL-1β: Interleukin-1β
